# Editorial: Case reports in autism

**DOI:** 10.3389/fpsyt.2024.1357823

**Published:** 2024-01-23

**Authors:** Marco Colizzi, Fengyu Zhang

**Affiliations:** ^1^ Unit of Psychiatry, Department of Medicine (DAME), University of Udine, Udine, Italy; ^2^ Global Clinical and Translational Research Institute, Bethesda, MD, United States

**Keywords:** autism, neurodevelopment, neuropsychiatry, treatment, diagnosis, behavior, comorbidity, environment

Autism is a neurodevelopmental disorder characterized by persistent deficits in social communication and interaction and restricted repetitive patterns of behaviors, interests, and activities (1). It has become an emerging condition that affects 2% of children under eight and throughout adulthood (2), partly due to improved performance of screening and diagnosis and public awareness. However, autism has great complexity and heterogeneity in etiology, clinical manifestations, and biological and behavioral features that cross multiple developmental domains, including physical, cognitive, social-emotional, and sensory, and there are limited options for treatment and intervention (3). Also, it is probably one of the most misunderstood conditions, where efforts are needed to increase comprehension of such a complex phenomenon and dispel myths and misconceptions that may hamper its identification and treatment (4). Case reports and focus groups allow an in-depth examination of individual cases to dissect this complexity and delineate the disease course and progression (4, 5). Even though case reports do not provide the most solid evidence-based indications, they may help perform exploratory analysis or generate intriguing hypotheses for higher-rank investigations in physiological studies and clinical trials (5).

This Research Topic reflects some current challenges and advances in autism. Research reports presented here approach autism for the complex condition that it is (6), thus ranging in their content and trying to offer an overview of the progress that has been made. Also, some evidence about emerging and promising therapeutic interventions is presented.

One of the significant interests in autism research is represented by the possibility of identifying autism as soon as possible, promptly intervening, and possibly modifying its trajectory positively (7). Malachowski et al. compared an infant with autism and a neurotypical infant regarding the early development of fine motor skills and visual attention to objects. Their study revealed how significant differences in such domains may emerge by just three months of age, possibly offering a behavioral marker for early detection of autism.

Another line of research is interested in investigating psychiatric comorbidity and outcomes among autistic children as they grow up (8). Di Luzio et al. presented a case where emerging psychotic symptoms along with late regression posed a diagnostic challenge whether clinicians were facing a very-early onset schizophrenia or autism where social-communicative development and adaptive functioning had been adequate during the first years of life. Authors reflected on the neurodevelopmental continuum paradigm, where autism and psychiatric disorders that emerge later in life can be conceptualized within a pattern of pathological continuity (9). This is particularly relevant for milder forms of autism and female individuals, whose attempt at camouflaging pathognomonic features of autism may exacerbate psychic distress. Secci et al. described a female adolescent whose undiagnosed autism represented a potential vulnerability for treatment-resistant depression with high suicide risk, possibly through exposure to stressful life events.

Some studies addressed strategies to sustain autistic children in everyday life and in times of difficulty (10). Nair et al. investigated autistic children’s preferences regarding colors and lighting to create autistic-friendly interior spaces. Evidence indicated calming or stimulating effects and behavioral changes, depending on hue, saturation, and luminosity, likely driven by atypical sensory processing. The authors emphasized how an autism-friendly built environment may support autistic people’s well-being and cognitive function. Arai et al. provided a valuable example of how collecting information on a child’s expected behavioral problems from parents and school teachers may be of support during hospital admission for surgery. Such an approach is of paramount importance for at least two main reasons. On the one hand, autistic children present with complex health needs, which make them more prone to any kind of hospital interactions; on the other, autistic children’s health management is challenging, as their difficulties render it more difficult to obtain compliance with healthcare pathways.

Following the last point, it is relevant to focus on medical comorbidity and its impact on autism presentation and outcome (11). Milutinovic et al. discussed a case of a child with autism who also presented with a genetic condition, the Coffin-Siris syndrome, that, along with physical abnormalities and dysfunctions, is characterized by neurocognitive and developmental difficulties. Interestingly, the identified heterozygous *de novo* pathogenic variant (class 5) c.1638_1647del in the *ARID1B* gene, which has a causative role in the Coffin-Siris syndrome, is *per se* associated with an autistic phenotype. Hu et al. focused on the *BRSK2* gene, which encodes the brain-specific serine/threonine protein kinase 2, the latter being independently implicated in autism pathophysiology. In such a case, the adolescent presenting with a *de novo* frameshift variant (c.442del, p.L148Cfs^*^39) suffered from attention-deficit symptoms, auditory hallucinations, and abnormal brain electrical activity mapping, thus revealing a severe form of the disorder. Such evidence urges an in-depth analysis of genetic causes and modifiers of autism (12).

Two studies have opened up novel biological therapies based on comorbid conditions (13). Offutt and Breitschwerdt provided evidence of how treating specific poly-microbial vector-borne infections resulted in overall neuropsychiatric improvement in an autistic adolescent. Interestingly, there was such an improvement that the patient dramatically increased his academic performance, moving from special education needs to grade-level standing without accommodations to college acceptance. The authors questioned whether there was a direct or indirect (i.e., secondary immune consequences) effect of the infections (i.e., bartonellosis and borreliosis) on autism development or, at least, specific phenotypic presentation. Hu et al., based on background supporting the role of the intestinal microbiota in autism, treated a child with fecal microbiota transplantation (FMT) from a healthy donor. FMT into the patient’s gastrointestinal tract improved the gut microenvironment, with effects not only for her gastrointestinal symptoms but also for autism core symptoms and overall functioning. The authors called for better-designed clinical trials of FMT and studies of the role of the microbiota in the pathophysiology of autism.

Some studies reported on non-pharmacological treatments, as well as environmental interventions, that may potentially address some unmet needs in the autistic population (14). Colombi et al. reported on the effects of a pre-emptive parent-mediated intervention based on the Infant Start, an adaptation of the Early Start Denver Model (ESDM), in an autistic child followed up during his very first months of life. Beneficial effects were detected on both the child’s developmental levels and autistic symptoms. Panesi et al. presented an intervention promoting working memory capacity and executive functions among pre-schooling autistic children by implementing digital apps and analogical playful activities. Interestingly, the child benefited from a nine-week intervention in terms of working memory-related language reception and update, inhibitory control, receptive vocabulary, and playful activities. Such findings highlight the importance of intervening in front of early warning signs of autistic behavior to sustain the child’s developmental trajectory. Maggio et al. presented a prospective case series exploring employment perspectives among autistic individuals who benefit from an individual-supported program to enhance placement in a sheltered work environment. Evidence indicated an improvement in working abilities and self-organization, despite patients having severe-to-moderate autism. The authors proposed to implement such intervention to enhance employment in autism with high support needs and co-occurring intellectual disability.

In summary, this Research Topic highlights how case reports could provide information on understudied aspects and emerging biobehavioral underpinnings of a condition, particularly rewarding for those conditions like autism, where the heterogeneity and complexity of phenotypes require the rapid acquisition of new data. We hope that the information summarized here could provide support for further study of new pathophysiological mechanisms and clinical management strategies.

## Author contributions

MC: Writing – original draft, Writing – review & editing. FZ: Writing – original draft, Writing – review & editing.
